# Postoperative pulmonary complications after totally thoracoscopic cardiac surgery: a retrospective cohort study comparing single-lumen and double-lumen endotracheal tubes

**DOI:** 10.3389/fmed.2026.1928907

**Published:** 2026-09-16

**Authors:** Rongquan Chen, Zhijin Hu, Yan Lin, Xiaoying Wu, Binlian Liu, Wenqing Zhang

**Affiliations:** 1 Department of Anesthesiology, Zhangzhou Municipal Hospital Affiliated with Fujian Medical University, Zhangzhou, Fujian, China; 2 Department of Orthopedics, Zhangzhou Municipal Hospital of Traditional Chinese Medicine, Zhangzhou, Fujian, China

**Keywords:** airway management, cardiac surgical procedures, intubation, one-lung ventilation, postoperative complications, thoracoscopy

## Abstract

**Objective:**

Double-lumen tubes (DLTs) are standard for one-lung ventilation in totally thoracoscopic cardiac surgery but are associated with airway injury and postoperative pulmonary complications (PPCs). This study compared perioperative outcomes between single-lumen tube (SLT)-based and DLT-based strategies in 176 adults undergoing elective totally thoracoscopic cardiac surgery (SLT, *n* = 94; DLT, *n* = 82).

**Methods:**

This was a single-center retrospective cohort study. The SLT-based strategy employed a single-lumen tube with two-lung ventilation supplemented by brief, controlled intermittent lung collapse when enhanced surgical exposure was required, whereas the DLT-based strategy employed a double-lumen tube with one-lung ventilation throughout the main phase of surgery. The primary outcome was PPC incidence; secondary outcomes included airway complications and recovery metrics. Multivariable and sensitivity analyses were performed.

**Results:**

Baseline characteristics were balanced. The anesthesia-to-incision time was shorter in the SLT group (57 vs. 67 min; *P* < 0.001). The SLT group had lower PPC incidence [29% vs. 56%; adjusted odds ratio (aOR), 0.30; 95% CI, 0.16–0.58], including lower rates of respiratory infection (7% vs. 22%) and atelectasis (18% vs. 39%). Airway complications were also less frequent (23% vs. 48%; aOR, 0.35; 95% CI, 0.18–0.68). Mechanical ventilation duration (17.5 vs. 21.0 h; *P* = 0.009) and ICU stay (28.0 vs. 36.0 h; *P* < 0.001) were shorter in the SLT group.

**Conclusion:**

An SLT-based strategy was associated with improved perioperative efficiency, fewer pulmonary and airway complications, and faster recovery. These findings warrant further investigation in prospective studies.

## 1 Introduction

Minimally invasive cardiac surgery (MICS) has emerged as a notable alternative to conventional median sternotomy, offering benefits such as reduced surgical trauma, less bleeding, shorter hospital stays, and faster recovery (1–4). Totally thoracoscopic cardiac surgery, a refined form of MICS, requires excellent surgical field exposure. The established standard method to achieve this is the use of a double-lumen endotracheal tube (DLT) to facilitate one-lung ventilation, in which the operative lung collapses (5, 6).

However, the use of a DLT poses several clinical challenges. For example, inserting a DLT is more technically demanding and time-consuming than inserting a single-lumen tube (SLT). Moreover, because of their relatively large diameter and complex structure, DLTs are associated with increased risks of airway trauma, postoperative hoarseness, and sore throat (7). Perhaps more critically, the one-lung ventilation process induces significant ventilation–perfusion mismatch, which can lead to hypoxemia and increase the risk of postoperative pulmonary complications (PPCs) due to increased mechanical stress and biotrauma (8, 9).

Within this context, the use of an SLT-based strategy involving two-lung ventilation represents a less invasive alternative for airway management. SLTs have a smaller outer diameter, facilitating easier and faster insertion and potentially reducing the risk of airway injury (10). In support of this approach, recent evidence suggests that two-lung ventilation with a low tidal volume and high respiratory frequency may confer lung-protective effects while maintaining adequate oxygenation and surgical conditions during thoracic surgery (11, 12).

Real-world data on patient-centered outcomes—such as postoperative pulmonary complications defined by standardized criteria and clinical resource utilization—remain limited. Therefore, this retrospective cohort study aims to complement existing evidence by evaluating patient-centered outcomes using standardized criteria in a real-world setting over a longer study period.

## 2 Materials and methods

### 2.1 Study design and patient selection

This was a single-center, retrospective cohort study approved by the Institutional Review Board of the participating institution (No: 2025LWB369); informed consent was waived.

All clinical data, including intraoperative variables, postoperative outcomes, and follow-up assessments, were extracted from the electronic medical records of our institution. These data were collected prospectively as part of routine clinical care and were extracted retrospectively for this study.

We consecutively screened all adults who underwent elective totally thoracoscopic cardiac surgery from December 2018 to July 2025. This time period was chosen because December 2018 marked the first use of SLT-based strategy at our institution, and July 2025 was the data extraction cutoff for this analysis. All eligible patients during this period were included as an exploratory cohort; no power calculation was performed prior to data collection.

### 2.2 Grouping and selection criteria

Patients were divided into two groups based on the type of endotracheal tube used for intubation: a single-lumen tube (SLT) group and a double-lumen tube (DLT) group. The choice of endotracheal tube was made by the attending anesthesiologist based on clinical judgment, not randomization. Factors that may have influenced this choice included anticipated surgical complexity, patient anatomy, and surgeon preference. This introduced potential selection bias, which is addressed in the limitations section.

The surgical procedures included mitral and tricuspid valve repair/replacement, closure of septal defects, and resection of atrial tumors.

The inclusion criteria were as follows: age ≥ 18 years; hemodynamically stable without inotropic support; scheduled for elective totally thoracoscopic cardiac surgery with cardiopulmonary bypass (CPB) requiring aortic cross-clamping; documented endotracheal tube type in the anesthetic record; and complete clinical data available for analysis, including key perioperative variables.

The exclusion criteria were as follows: emergency surgery; preoperative hemodynamic instability requiring inotropic support; left ventricular ejection fraction < 30%; dialysis-dependent renal failure or severe liver disease; severe anemia (preoperative hematocrit < 20%); previous cardiac or thoracic surgery; conversion to sternotomy; preexisting difficult airway or severe chronic respiratory disease; pregnancy; and incomplete clinical records for key perioperative variables.

### 2.3 Anesthesia and surgical management

#### 2.3.1 Anesthesia management

Standard monitoring (including electrocardiogram, pulse oximetry, and noninvasive blood pressure monitoring) was performed upon arrival in the operating room. Invasive arterial pressure monitoring was achieved via the use of radial artery cannulation under local anesthesia. General anesthesia was induced with midazolam (0.02–0.08 mg/kg), sufentanil (1–5 μg/kg), propofol (1–2.5 mg/kg), and cisatracurium (0.2–0.4 mg/kg). In all cases, endotracheal intubation was performed using a video laryngoscope (model: VL310–4; UE Medical, China). The position of the tube in the SLT group was confirmed via lung auscultation. In the DLT group, a left-sided double-lumen tube (Smiths Medical, USA) was used. After passage through the vocal cords, it was advanced blindly into the mainstem bronchus. Tube position was initially assessed by auscultation to confirm lung isolation, followed by fiberoptic bronchoscopy for precise positioning of the bronchial lumen in the left mainstem bronchus. According to the available anesthesia records, all patients in the DLT group were successfully intubated with a left-sided DLT. No case requiring conversion to a right-sided DLT due to anatomic variations or placement failure was documented. Anesthesia was maintained with continuous infusions of propofol (2–4 mg·kg⁻^1^·h⁻^1^), remifentanil (0.4 μg·kg⁻^1^·min⁻^1^), cisatracurium (0.1–0.2 mg·kg⁻^1^·h⁻^1^), dexmedetomidine (0.5 μg·kg⁻^1^·h⁻^1^), and inhaled sevoflurane (1–3 vol%), with the goal of targeting a bispectral index between 40 and 60.

#### 2.3.2 Ventilation management

In this study, the two groups represented distinct airway and ventilation strategies rather than merely different endotracheal tubes. The SLT-based strategy employed a single-lumen tube with two-lung ventilation, supplemented by brief, controlled intermittent lung collapse (≤ 2 min) when improved surgical exposure was required. The DLT-based strategy employed a double-lumen tube with one-lung ventilation throughout the main phase of surgery.

### 2.4 SLT group

An SLT (Beter, China) was used in the SLT group. Two-lung ventilation was intraoperatively maintained with the following ventilator settings: tidal volume of 4–6 mL·kg^−1^ (ideal body weight), respiratory rate of 12–16 breaths·min^−1^, fraction of inspired oxygen (FiO_2_) of 50%–100%, and positive end-expiratory pressure (PEEP) of 0–5 cmH_2_O. During critical surgical steps requiring improved exposure (e.g., port placement or cardiac manipulation), intermittent lung collapse was performed after discussion with the surgeon. Under controlled conditions and using aseptic techniques, the ventilator circuit was briefly disconnected to allow the collapse of the operative lung for ≤ 2 min under thoracoscopic visualization. Peripheral oxygen saturation (SpO_2_) was continuously monitored throughout the procedure; a predefined intervention criterion of SpO_2_ < 90% prompted a prompt resumption of ventilation and corrective measures (e.g., increasing the FiO_2_, temporarily returning to two-lung ventilation, and recruitment maneuvers). During CPB, low-frequency two-lung ventilation was maintained (tidal volume: 4 mL·kg^−1^; respiratory rate: 6 breaths·min^−1^; FiO_2_: 50%; PEEP: 0–5 cmH_2_O) to maintain alveolar patency and reduce postoperative atelectasis.

### 2.5 DLT group

In this group, left-sided one-lung ventilation was implemented during the main phase of surgery (defined as the period from initiation of one-lung ventilation after patient positioning until chest closure, excluding the CPB period). Ventilator parameters during this phase were as follows: Tidal volume of 4–6 mL/kg, respiratory rate of 12–18 breaths/min, FiO₂ of 50%–100%, and PEEP of 0–5 cmH₂O. The use of PEEP 0–5 cm H₂O is standard practice at our institution for one-lung ventilation during thoracoscopic surgery. During CPB, the DLT was disconnected from the ventilator, and the lungs were left deflated. No mechanical ventilation was delivered during this period. After CPB, one-lung ventilation was resumed until chest closure. After chest closure, the DLT was exchanged for an SLT in the operating room to facilitate postoperative mechanical ventilation and airway management in the intensive care unit (ICU).

#### 2.5.1 Surgical procedure

All of the operations were performed via the use of a standardized totally thoracoscopic three-port technique (13). The patients were positioned supine with their right side elevated 15–20°, and external defibrillation electrodes were routinely attached to the skin. CPB was established via femoral arterial and venous cannulation.

### 2.6 Study outcomes

The primary outcome was the incidence of PPCs, as defined according to the European Perioperative Clinical Outcome criteria (14, 15). This composite outcome included the following conditions: Respiratory infection, respiratory failure, pleural effusion, atelectasis, pneumothorax, bronchospasm, and aspiration pneumonitis. Detailed operational definitions for each component, the observation period, and the handling of intraoperative atelectasis were provided in Supplementary Material.

The following secondary outcomes were analyzed: Postoperative airway complications, a composite outcome defined as the presence of either hoarseness or sore throat within the first postoperative week. Sore throat was assessed using a 0–100 mm visual analog scale (VAS; 0 = no pain, 100 = worst pain imaginable), with a score >30 mm defined as a clinically relevant sore throat (16). Hoarseness was graded using a 4-point scale: 0 = no complaint of hoarseness; 1 = minimal change in voice quality (mild hoarseness); 2 = moderate change in voice quality (moderate hoarseness); 3 = severe change in voice quality (severe hoarseness) (17). For the primary composite outcome, hoarseness was considered present only if the patient reported at least moderate hoarseness (grade ≥ 2). Both sore throat VAS scores and patient-reported hoarseness were routinely documented in daily postoperative follow-up notes at our institution. The incidence of the composite outcome was recorded as ‘yes’ if either condition was present; surgery-related metrics, which included the time from anesthesia induction to skin incision (I-S time), total anesthesia time, total operative time, CPB time and aortic cross-clamp (ACC) time; and recovery profiles, which included the duration of postoperative mechanical ventilation, length of ICU stay, postoperative 24-hour chest tube drainage volume, postoperative hospital stay, and 30-day mortality.

### 2.7 Statistical analysis

Statistical analyses were performed using IBM SPSS Statistics for Windows, version 25.0 (IBM Corp., Armonk, NY, USA). Categorical variables are presented as *n* (%) and were compared using the *χ*^2^ test or Fisher's exact test. Normality of continuous variables was assessed using the Shapiro–Wilk test. Normally distributed variables are presented as mean ± SD and were compared using the independent samples t test; non-normally distributed variables are presented as median [IQR] and were compared using the Mann–Whitney U test. The standardized mean difference (SMD) was calculated as the difference in means divided by the pooled standard deviation. Unadjusted effect estimates [odds ratios for binary outcomes; regression coefficients (β) for continuous outcomes] are presented with 95% confidence intervals derived from simple logistic or linear regression models. Multivariable analyses were performed for outcomes with >10 events using logistic or linear regression, adjusting for age, smoking history, body mass index, and ASA physical status. A sensitivity analysis additionally adjusted for CPB time. As this was an exploratory analysis of all eligible cases during the study period, no prospective sample size calculation was performed. Two-tailed *P* < 0.05 was considered statistically significant.

## 3 Results

### 3.1 Baseline characteristics

Of the 212 patients screened, 176 met the inclusion criteria (Figure 1): 94 in the SLT group and 82 in the DLT group. Baseline demographics, comorbidities, and perioperative risk factors were well balanced between the two groups (Table 1). The proportion of SLT-based strategy use increased from 48% in the early period (2018–2021) to 61% in the later period (2022–2025) (Supplementary Table S2).

**Figure 1:**
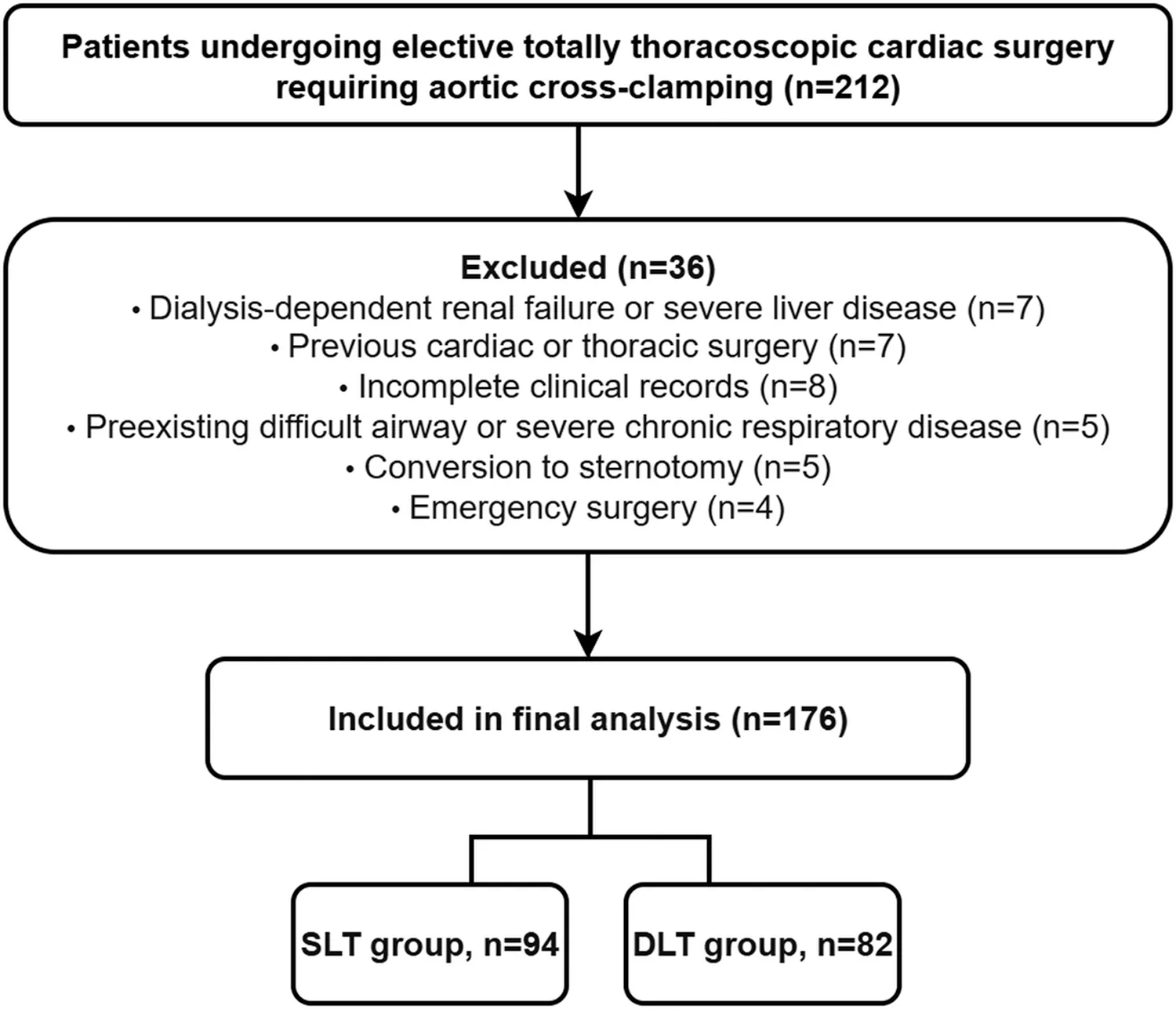
Flow diagram of patient selection.

**Table 1:** Baseline patient characteristics before surgery.

Variable	SLT group (*N* = 94)	DLT group (*N* = 82)	*P* value	SMD (%)
Female, *n/N* (%)	56/94 (60)	48/82 (59)	> 0.999	2.1
Age (yr)	55 [43–66]	57 [49–63]	0.646	13.1
Body mass index (kg/m^2^)	22.0 [20.8–24.3]	22.3 [20.3–24.2]	0.944	3.0
Hemoglobin (g/L)	127 ± 25	129 ± 22	0.627	7.4
LVEF (%)	60.8 ± 7.4	61.5 ± 7.5	0.579	8.4
ASA physical status, *n/N* (%)			0.503	17.7
II	27/94 (29)	30/82 (37)		
III	63/94 (67)	48/82 (58)		
IV	4/94 (4)	4/82 (5)		
Surgical Type, *n* (%)			0.957	
ASD/VSD	23 (24.5)	22 (26.8)		7.3
MVP/MVR	55 (58.5)	45 (54.9)		5.4
TVP/TVR	11 (11.7)	11 (13.4)		5.1
Cardiac Tumor Resection	5 (5.3)	4 (4.9)		2.0
Hypertension, *n/N* (%)	27/94 (29)	18/82 (22)	0.393	15.6
Diabetes mellitus, *n/N* (%)	5/94 (5)	5/82 (6)	> 0.999	3.4
Coronary heart disease, *n/N* (%)	15/9 4 (16)	12/82 (15)	0.973	3.7
Cerebrovascular disease, *n/N* (%)	8/94 (9)	7/82 (9)	> 0.999	1.0
Atrial fibrillation, *n/N* (%)	11/94 (12)	9/82 (11)	> 0.999	2.3
Smoking history, *n/N* (%)	15/94 (16)	16/82 (20)	0.675	9.3

ASA, American Society of Anesthesiologists; ASD, atrial septal defect; DLT, double-lumen tube; LVEF, left ventricular ejection fraction; MVP, mitral valve plasty; MVR, mitral valve replacement; SLT, single-lumen tube; SD, standard deviation; IQR, interquartile range; SMD, standardized mean difference; TVP, tricuspid valve plasty; TVR, tricuspid valve replacement; VSD, ventricular septal defect. An SMD < 0.2 is considered to indicate acceptable balance between groups.

### 3.2 Postoperative outcomes

#### 3.2.1 Pulmonary and airway complications

The incidence of PPCs was significantly lower in the SLT group than the DLT group (29% vs. 56%; OR, 0.32; 95% CI, 0.17 to 0.59; *P* < 0.001), with lower incidences of respiratory infection (7% vs. 22%; OR, 0.29; 95% CI, 0.11 to 0.73; *P* = 0.011) and atelectasis (18% vs. 39%; OR, 0.35; 95% CI, 0.17 to 0.69; *P* = 0.003). Airway complications including hoarseness and sore throat were also less frequent in the SLT group (23% vs. 48%; OR, 0.29; 95% CI, 0.14 to 0.59; *P* = 0.001). The incidence of other individual pulmonary and airway events did not significantly differ between the groups (Table 2).

**Table 2:** Postoperative outcomes: unadjusted comparisons.

Variable	SLT group (*n* = 94)	DLT group (*n* = 82)	Unadjusted effect (95% CI)	*P* value
Pulmonary complications, *n* (%)	27 (29)	46 (56)	0.32 (0.17–0.59)	**<** **0.001**
Respiratory infection, *n* (%)	7 (7)	18 (22)	0.29 (0.11–0.73)	**0**.**011**
Respiratory failure, *n* (%)	3 (3)	3 (4)	0.87 (0.17–4.48)	> 0.999
Pleural effusion, *n* (%)	16 (17)	15 (18)	0.92 (0.42–2.00)	0.982
Atelectasis, *n* (%)	17 (18)	32 (39)	0.35 (0.17–0.69)	**0**.**003**
Pneumothorax, *n* (%)	5 (5)	5 (6)	0.87 (0.24–3.13)	> 0.999
Bronchospasm, *n* (%)	2 (2)	2 (2)	0.87 (0.12–6.40)	> 0.999
Aspiration pneumonitis, *n* (%)	2 (2)	2 (2)	0.87 (0.12–6.40)	> 0.999
Airway complications, *n* (%)	22 (23)	39 (48)	0.29 (0.14–0.59)	**0**.**001**
Hoarseness, *n* (%)	14 (14.9)	27 (32.9)	0.36 (0.17–0.74)	**0**.**008**
Sore throat, *n* (%)	10 (10.6)	23 (28.1)	0.31 (0.14–0.69)	**0**.**006**
24-h drainage (mL)	180 ± 79	175 ± 67	4.77 (−17.24 to 26.77)	0.669
Mechanical ventilation (h)	17.5 [14.0–23.8]	21.0 [15.0–34.0]	−5.15 (−8.92–−1.39)	**0**.**009**
ICU stay (h)	28.0 [21.0–40.0]	36.0 [29.0–48.8]	−7.06 (−11.47–−2.64)	**<** **0.001**
hospital stay (d)	10 [8–12]	11 [8–14]	−0.83 (−1.96–0.31)	0.163
Mortality, *n* (%)	2 (2)	1 (1)	1.76 (0.15–20.12)	> 0.999

OR, odds ratio; β, regression coefficient; CI, confidence interval; DLT, double-lumen tube; ICU, intensive care unit; SLT, single-lumen tube. Bold values indicate statistical significance (*p* < 0.05).

#### 3.2.2 Recovery and other outcomes

Patients in the SLT group exhibited a shorter duration of mechanical ventilation (median, 17.5 h vs. 21.0 h; *β* = −5.15; 95% CI, −8.92 to −1.39; *P* = 0.009) and a shorter ICU stay (median, 28.0 h vs. 36.0 h; *β* = −7.06; 95% CI, −11.47 to −2.64; *P* < 0.001). No significant differences were observed in 24-h chest tube drainage, postoperative hospital stay, or 30-day mortality (Table 2).

#### 3.2.3 Multivariable and sensitivity analyses

After adjusting for age, smoking history, body mass index, and ASA status, SLT use remained independently associated with lower odds of PPCs and airway complications, and shorter ICU stays (Table 3). A sensitivity analysis with additional adjustment for CPB time showed a decrease in the odds ratio for PPCs, while estimates for other outcomes remained stable (Supplementary Table S1).

**Table 3:** Multivariable analysis of postoperative outcomes (primary model).

Variable	Events, n	aOR/β (95% CI)^a^
Pulmonary complications	73	0.30 (0.16–0.58)
Respiratory infection	25	0.25 (0.11–0.74)
Pleural effusion	31	1.11 (0.46–2.67)
Atelectasis	49	0.30 (0.13–0.68)
Airway complications	61	0.35 (0.18–0.68)
Hoarseness	41	0.37 (0.18–0.80)
Sore throat	33	0.29 (0.13–0.68)
24-h drainage (mL)	176	5.58 (−16.59–27.74)
Mechanical ventilation (h)	176	−5.13 (−8.96–−1.31)
ICU stay (h)	176	−7.34 (−11.79–−2.90)
hospital stay (d)	176	−0.95 (−2.07–0.17)

a Adjusted for age, smoking history, body mass index, and ASA physical status. aOR, adjusted odds ratio; β, regression coefficient; CI, confidence interval; ICU, intensive care unit.

### 3.3 Intraoperative outcomes

The anesthesia-to-skin incision time was significantly shorter in the SLT group than in the DLT group (median, 57 min vs. 67 min; *β* = −7.84; 95% CI, −10.67 to −5.01; *P* < 0.001). No significant between-group differences were observed in total anesthesia time, operative time, cardiopulmonary bypass time or aortic cross-clamp time (Table 4).

**Table 4:** Intraoperative data and outcomes.

Variable	SLT group (*n* = 94)	DLT group (*n* = 82)	Unadjusted difference (95% CI)	*P* value
I-S time (min)	57 [53–65]	67 [60–73]	β −7.84 (−10.67–−5.01)	**<** **0.001**
Anesthesia time (min)	341 [297–372]	348 [321–372]	β −8.09 (−21.–5.71)	0.241
Operative time (min)	276 [229–305]	270 [243–289]	β 5.34 (−8.–19.41)	0.557
CPB time (min)	172 ± 25	166 ± 26	β 5.87 (−1.62–13.37)	0.124
ACC time (min)	110 ± 23	110 ± 24	β 0.09 (−7.12–6.93)	0.979

OR, odds ratio; β, regression coefficient; CI, confidence interval; ACC, aortic cross-clamp; CPB, cardiopulmonary bypass; DLT, double-lumen tube; I-S time, induction of anesthesia-to-skin incision time; SLT, single-lumen tube. Bold values indicate statistical significance (*p* < 0.05).

## 4 Discussion

In this retrospective cohort study of patients undergoing totally thoracoscopic cardiac surgery, we observed that compared with a DLT-based strategy, an SLT-based strategy with intermittent lung collapse was associated with faster preoperative preparation, fewer postoperative pulmonary and airway complications, and accelerated recovery. These findings suggest potential benefits of simplified airway management in the perioperative setting. This study was retrospective and hypothesis-generating; the observed associations should not be interpreted as causal, but rather as signals to guide future prospective research.

The observed reduction in the anesthesia-to-skin incision time is consistent with previous reports and reflects the fact that SLT insertion is simpler than DLT insertion and that DLT positioning but not SLT positioning requires routine fiberoptic bronchoscopy (11, 12). Although this time advantage did not extend to the total anesthesia or operative time—likely due to the fixed sequence of surgical steps and consistent team performance, which are the primary determinants of total anesthesia duration—it nonetheless contributed to a more streamlined preoperative workflow. More importantly, this simpler approach may have facilitated the adoption of lung-protective ventilation strategies, which may have contributed to the lower incidence of postoperative pulmonary complications (PPCs) observed in the SLT group.

The observed difference in the composite PPC endpoint was driven largely by lower rates of respiratory infection and atelectasis in the SLT group, with a 43% relative reduction in overall PPC incidence. This finding supports the hypothesis that maintaining two-lung ventilation for most of the procedure may attenuate ventilation-induced lung injury. Prolonged one-lung ventilation with a DLT is known to induce ventilation–perfusion mismatch, ischemia–reperfusion injury, and regional mechanical stress (18–21). In contrast, the SLT-based strategy maintained near-physiological two-lung ventilation for most of the procedure, with brief, controlled lung collapse occurring only during critical steps, which may have better preserved alveolar stability and mucociliary clearance. Importantly, the SLT-based strategy maintained two-lung ventilation for most of the procedure, with brief, controlled interruptions when improved surgical exposure was required, including during CPB. Accumulating evidence suggests that maintaining ventilation during CPB—even with low tidal volumes—preserves lung compliance, reduces the risk of atelectasis, and attenuates systemic inflammation (22–25). However, most of this evidence comes from studies of conventional sternotomy cardiac surgery, and caution is warranted when extrapolating to totally thoracoscopic procedures. After additional adjustment for CPB time, the adjusted odds ratio for PPCs decreased from 0.30 to 0.18. Whether CPB time acts as a confounder or a mediator cannot be determined from these data.

Nevertheless, our findings are consistent with those of a recent randomized trial by Wang et al. (11), which also reported fewer airway complications and lower atelectasis rates with SLT. Extending beyond that trial, our study additionally showed that the SLT-based strategy was associated with shorter durations of mechanical ventilation and ICU stay, outcomes not assessed in the randomized trial. The convergence of directional signals across a randomized controlled trial and a real-world cohort suggests that the potential benefits of an SLT-based strategy warrant further investigation in prospective studies.

The SLT-based strategy was also associated with fewer airway complications, including hoarseness and sore throat. This advantage is consistent with two factors: the smaller outer diameter and more compliant material of SLTs, and the avoidance of routine DLT-to-SLT exchange under deep anesthesia at the end of surgery (11). The DLT-to-SLT exchange represents an additional airway instrumentation that may itself cause vocal cord edema, laryngeal mucosal injury, and postoperative hoarseness or sore throat.

The combined benefits of reduced pulmonary and airway morbidity likely contributed to the shorter durations of mechanical ventilation and ICU stay observed in patients in the SLT group. Patients with fewer complications require less intensive respiratory support and may be weaned from ventilation earlier (26). These improvements are clinically relevant, as even modest reductions in ICU length of stay may translate into important resource savings and enhanced patient recovery.

This study has several limitations. First, its retrospective, non-randomized design and the seven-year enrollment period may introduce selection bias and temporal confounding. Second, the two strategies differ in both tube type and ventilation approach, which limits the ability to attribute the observed benefits solely to the tube. Third, the cumulative duration of intermittent lung collapse in the SLT group was not quantified, and standardized measures of surgical field exposure were not collected, limiting mechanistic interpretation. Future prospective studies should include surgeon-rated exposure quality as a key outcome. We also acknowledge that differentiating respiratory infection from atelectasis in the postoperative period can be challenging in a retrospective study, and some degree of misclassification cannot be excluded despite the operational criteria applied. Additional limitations include model instability after adjustment for CPB time, the unavailability of preoperative pulmonary function data, and the lack of adjustment for procedure type in the multivariable model. Despite these limitations, this real-world study offers hypothesis-generating evidence that complements existing randomized trial data.

In conclusion, this retrospective cohort study suggests that an SLT-based strategy with intermittent lung collapse may be associated with faster preoperative preparation, fewer pulmonary and airway complications, and accelerated recovery. The observed associations should be confirmed in future prospective studies, which would also help identify specific patient subgroups—particularly those at highest risk for PPCs—who would derive the greatest benefit from this approach.

## Data Availability

The raw data supporting the conclusions of this article will be made available by the authors, without undue reservation.
