# Supplementary material for: Postoperative pulmonary complications after totally thoracoscopic cardiac surgery: a retrospective cohort study comparing single-lumen and double-lumen endotracheal tubes
Source: Front Med (Lausanne). 2026 Sep 16;13:1928907. doi: 10.3389/fmed.2026.1928907 (PMC13624132; doi:10.3389/fmed.2026.1928907)
Supplement: Supplementary file 1 [file Datasheet1.docx]

Supplementary Material

Detailed definitions of postoperative pulmonary complications (PPCs)

The definitions were based on the European Perioperative Clinical Outcome criteria, with operational refinements for retrospective data ascertainment as follows:

Respiratory infection: clinical signs (fever ≥ 38°C, leukocytosis > 12,000/μL) plus positive sputum culture or new infiltrate on chest X-ray, requiring antibiotic treatment for ≥ 7 days.

Respiratory failure: was defined as the unplanned need for noninvasive or invasive ventilation after initial extubation, reintubation, or mechanical ventilation for more than 48 hours post-operatively.

Pleural effusion: was defined as new or increased pleural effusion on postoperative chest imaging that required therapeutic intervention (e.g., thoracentesis or chest tube drainage).

Atelectasis: New-onset lung opacification on chest X-ray with volume loss (displacement of fissures or the mediastinum) confirmed by a radiologist.

Pneumothorax: air in pleural space on chest X-ray.

Bronchospasm: wheezing requiring bronchodilator treatment.

Aspiration pneumonitis: is characterized by the aspiration of gastric contents with subsequent hypoxemia or radiographic changes.

The observation period was from the end of surgery until hospital discharge or postoperative day 30, whichever occurred first.

Note: Atelectasis was considered a PPC only if present postoperatively; intraoperative atelectasis (expected during one-lung ventilation) was not included in the primary outcome.

Differentiation between respiratory infection and atelectasis

In this retrospective analysis, the distinction between respiratory infection and atelectasis was based on the information available in the medical records. The following operational criteria were applied to standardize the classification:

• Respiratory infection was assigned when the medical records documented positive sputum culture or new infiltrate on chest X-ray accompanied by clinical signs of infection (fever ≥ 38°C, leukocytosis > 12,000/μL) and initiation of antibiotic treatment for ≥ 7 days.

• Atelectasis was assigned when new-onset lung opacification with volume loss was reported on chest X-ray (confirmed by a radiologist) and there was no documented evidence of infection (e.g., negative sputum culture, no antibiotic initiation, or resolution with respiratory therapy alone).

• When both conditions coexisted (e.g., atelectasis with superimposed infection), the case was classified as having respiratory infection if the criteria for infection were met; otherwise, it was classified as atelectasis.

TABLE S1 Sensitivity analysis with additional adjustment for the CPB time

| Variable | Events, n | Primary adjusted model aOR/β (95% CI)^*^ | Sensitivity analysis aOR/β (95% CI)^†^ | Change in estimate, %^‡^ |
| --- | --- | --- | --- | --- |
| Pulmonary complications | 73 | 0.30 (0.16 to 0.58) | 0.18 (0.07 to 0.46) | -41 |
| Respiratory infection | 25 | 0.25 (0.11 to 0.74) | 0.26 (0.09 to 0.81) | 7 |
| Pleural effusion | 31 | 1.11 (0.46 to 2.67) | 1.15 (0.48 to 2.79) | 4 |
| Atelectasis | 49 | 0.30 (0.13 to 0.68) | 0.29 (0.13 to 0.66) | -4 |
| Airway complications | 61 | 0.35 (0.18 to 0.68) | 0.35 (0.18 to 0.69) | 1 |
| Hoarseness | 41 | 0.37 (0.18 to 0.80) | 0.35 (0.16 to 0.75) | -7 |
| Sore throat | 33 | 0.29 (0.13 to 0.68) | 0.31 (0.13 to 0.73) | 7 |
| 24-h drainage (mL) | 176 | 5.58 (-16.59 to 27.74) | 6.59 (-15.74 to 28.92) | 18 |
| Mechanical ventilation (h) | 176 | -5.13 (-8.96 to -1.31) | -5.20 (-9.06 to -1.34) | -1 |
| ICU stay (h) | 176 | -7.34 (-11.79 to -2.90) | -7.48 (-11.96 to -3.00) | -2 |
| Hospital stay (d) | 176 | -0.95 (-2.07 to 0.17) | -0.95 (-2.08 to 0.18) | 0 |

* Adjusted for age, smoking history, body mass index, and ASA physical status.

† Additionally, adjusted for CPB time.

‡ Change in estimate: calculated as (sensitivity analysis point estimate – primary model point estimate)/∣primary model point estimate∣ × 100%.

aOR = adjusted odds ratio; β = regression coefficient; CI = confidence interval; CPB = cardiopulmonary bypass; ICU = intensive care unit.

TABLE S2 Temporal distribution of SLT and DLT use

| Period | SLT group, n (%) | DLT group, n (%) | Total, n |
| --- | --- | --- | --- |
| 2018–2021 | 48 (48) | 53 (52) | 101 |
| 2022–2025 | 46 (61) | 29 (39) | 75 |
| Total | 94 (53) | 82 (47) | 176 |
